# Yellow fever in the Americas: the growing concern about new epidemics

**DOI:** 10.12688/f1000research.11280.2

**Published:** 2017-04-25

**Authors:** Yeimer Ortiz-Martínez, Andrés Mauricio Patiño-Barbosa, Alfonso J. Rodriguez-Morales

**Affiliations:** 1 Universidad de Sucre, Sincelejo, Sucre, Colombia; 2 Public Health and Infection Research Group, Faculty of Health Sciences, Universidad Tecnológica de Pereira, Pereira, Risaralda, Colombia; 3 Colombian Collaborative Network on Zika and other Arboviruses (RECOLZIKA), Pereira, Risaralda, Colombia

**Keywords:** yellow fever, epidemics, Africa, Americas, Brazil, vector-borne disease, arbovirus

## Abstract

Yellow fever (YF) is a haemorrhagic viral disease with a high case fatality rate. It is considered a reemerging infectious disease of remarkable importance. During the last outbreaks in Brazil (2016-2017), many cases of YF emerged despite high YF vaccination coverage in some areas. However, there are many areas and populations worldwide where vaccination coverage has been low for years (e.g. Nigeria), which increases the risk of major epidemics in such areas, as would be the case in many of the American territories. Several factors, including the vast border and migratory status of Brazil, the widespread distribution of *Aedes* mosquitoes and the lack of efficient health policies and surveillance systems, favor this complex epidemiological scenario of reemergence. Therefore, mass vaccination of the population at risk, public health awareness and preparedness are urgently needed in this region. This opinion article describes the current global epidemiological situation of YF, focusing especially on the Americas, as well the risk and vulnerabilities in the region that would be of concern for major expansion to other countries apart from Brazil. Also, imported risk from endemic area outside of Americas (i.e. Africa) are of current concern.

## Introduction

Yellow fever (YF) is a haemorrhagic viral, vector-borne disease with a high case fatality rate (CFR), spread by infected mosquitoes caused by the YF virus, an arbovirus belonging to the genus Flavivirus, family Flaviviridae. It has reappeared as a threat to global public health, evidenced by new epidemics in several countries in Africa and South America through autochthonous transmission, and in Asia with imported cases ^1^. In Asia, but also Europe and North America, potential spreads beyond the borders of the endemic countries is a matter of global concern. Currently, there are around 1 billion people, from 49 endemic countries, that are considered at risk ^1, 2^. In this opinion article, we would like to express our concern regarding the expansion of YF in Latin America, beyond Brazil. In that country there is currently an epidemic situation, where, since the beginning of the outbreak in December 2016 up to 29 March 2017, there have been 1,987 cases of yellow fever reported (574 confirmed, 926 discarded, and 487 suspected under investigation). This included 282 deaths (187 confirmed, 24 discarded, and 71 under investigation) with a CFR of 33% among confirmed cases.

## Recent outbreaks outside the Americas

Although relatively wide scale YF vaccination has been applied, a growing number of outbreaks have been documented in several African countries in the last decade ^1– 3^. The most recent outbreak occurred in Angola, resulting in 7,344 suspected cases, 962 laboratory-confirmed cases and 137 deaths (with a CFR of 14.2%), and lasting from December 2015 to October 2016 ^2^. In addition to spread of YF by autochthonous transmission, confirmed imported cases of YF were identified in China and Kenya ^1– 3^. Other countries, such as Chad, Ghana and Guinea have also reported outbreaks or sporadic cases not linked to the outbreak in Angola ^1– 15^. On Mar 12, 2016, the first imported case of YF was confirmed by China CDC. The patient was a 32 years old Chinese male who worked in Luanda and had fever and chills on Mar 8, 2016. He arrived to Beijing, where he was immediately hospitalized. Eleven imported cases of confirmed yellow fever occurred in China from March of 2016, prompting calls to strengthen surveillance systems at the border ( http://www.who.int/csr/don/6-april-2016-yellow-fever-china/en/) ^16^.

## The risk of Yellow Fever in the Americas, with a focus on Brazil

Even though no new cases have been confirmed since the last year in Angola, Africa, the global threat continues, indeed now with its epicentre in Brazil, South America. An ongoing outbreak of YF has started in Brazil since December 1, 2016. Up to February 22, 2017, a total of 1,336 cases of YF infection have been reported (292 laboratory confirmed, 920 suspected and 124 ruled out), resulting in 215 deaths (101 confirmed, 109 suspected, 5 ruled out) across six states of the country (Bahia, Espírito Santo, Minas Gerais, Rio Grande do Norte, São Paulo and Tocantins). The current CFR is 35% (from confirmed cases) and 12% (from suspected cases) ^3^.

The geographical spread of the cases in Brazil has led to major concern, because cases are no longer being reported just in the jungle, but also in the most densely populated cities and states such as Minas Gerais and São Paulo. Fortunately, these regions have a long history of high YF vaccination coverage in young people (at least in urban areas), in contrast with the low vaccination rates in other major urban centres of Brazil ^4^. Nevertheless, the lack of YF vaccination coverage was raised several times by the Brazilian provincial health authorities back in the early 2000s, but they were unable to reach the remote western zones of Minas Gerais.

Although the epidemiology and clinical manifestations of YF should be familiar to healthcare workers in endemic countries, where clinical manifestations can overlap with other acute viral haemorrhagic fevers and other etiologies of the febrile syndrome, a rapid spread of misinformation about this harmful disease in social media and a lack of online training for healthcare workers has been reported in the recent outbreak of 2016–2017 in the Americas ^5, 6^. In addition to limited health resources, this highlights that early identification could be a challenge in Latin America, as has been observed in the past with Zika and chikungunya virus outbreaks in this region, particularly in countries such as Brazil and Colombia ^7, 8^.

Given the current YF situation in Brazil, and the emergence of new cases in areas where YF has not occurred for several years, the Brazilian health policies have been oriented to continue efforts to detect, confirm, and adequately and timely treat cases of YF ( http://portalsaude.saude.gov.br/index.php/o-ministerio/principal/secretarias/svs/febre-amarela). To this end, health care workers should be kept up to date and trained to detect and treat cases, especially in areas of known virus circulation. Furthermore, they should also take the necessary actions to keep travelers, heading to areas where YF vaccination is mandatory, informed and vaccinated.

## Conclusions

There seems to be an almost imminent risk of YF outbreaks turning into a large epidemic ^9^. Unvaccinated travelers heading to the affected states in Brazil are at risk of spreading the virus in to areas where YF risk factors (human susceptibility, prevalence of competent vector, and animal reservoirs) are present. The geographic area of human cases has expanded recently to Rio de Janeiro, São Paulo, and Pará states. Ecological factors and enzootics would promote the necessary spillover that would lead to an epidemic ^10– 12^, and unfortunately, nothing can be done to halt the infection of non-human primates in the affected areas. Moreover, the vast border of Brazil, with 10 neighboring countries/territories (Uruguay, Argentina, Paraguay, Bolivia, Peru, Colombia, Venezuela, Guyana, Suriname and French Guiana), the lack of efficient health policies and surveillance systems, and the distribution of *Aedes* vectors (as well the uncontrollable sylvatic vector species in the genus *Haemagogus* and *Sabethes*), raise the possibility of the widespread YF throughout the Americas, including the USA. The USA has suitable conditions for autochthonous cases in areas such as South Florida, where *Aedes albopictus* is present and has been linked to transmission of dengue virus (another flavivirus), chikungunya and possibly Zika. These entomological factors should strongly emphasize the risk of imported cases in temperate zones (i.e. Central and North Americas) during the boreal summer and *Aedes spp*. activity in Central and North America. It has been documented elsewhere for Dengue virus (New Mexico or Texas) and, even in the case of vectors for other diseases, such as *Anopheles*, the case of airport malaria in the US.

### The risk of imported cases in temperate zones

Mass vaccination of the at-risk population ^13^, and public health awareness and preparedness is urgently needed to control the current 2016–2017 outbreak in Brazil and prevent a possible epidemic related to this deadly disease. Nevertheless, these recent outbreaks and the lack of YF vaccine stock piling (WHO) means that YF vaccine availability needs to be strategized by the countries health authorities and international community. Also, the lifelong protection of the vaccine, its innocuity and the reduction by 1/10 of the immunity dose are new and of extremely high importance for global and public health.

More studies, as well as new innovative strategies for vector control (e.g. involving community participation), early prevention (e.g. sampling in risk areas to look for asymptomatic subjects), warning and enhanced surveillance (using smart phones), are necessary in order to improve the scenario of this reemerging arboviral threat ^14, 15^. Mosquito biosurveillance is an important issue to control the epidemic risk. *Haemagogus* and *Sabethes* are specific for South America and have been well studied; the risk and ability of *Aedes albopictus* expansion to transmit the virus in the Americas needs to be assessed, and an entomological priority set up when needed (i.e. public health priority in at-risk areas).

Finally, another matter of concern that is also important to mention is the trans-border risk. Ultimately traveler’s to/from endemic areas need to be covered by a mandatory international certificate of vaccination to protect the borders (trans-border risk). The long-time mystery of the absence of YF in South East Asia up to 2016 is also an issue to keep in mind for the future - particularly in term of global risk, with new imported cases, for example those reported in China, now occurring.
