# Supplementary material for: Yellow fever in the Americas: the growing concern about new epidemics
Source: F1000Res. 2017 Apr 25;6:398. Originally published 2017 Mar 30. [Version 2] doi: 10.12688/f1000research.11280.2 (PMC5414809; doi:10.12688/f1000research.11280.2)
Supplement: Peer review history

# Peer review: Referee response for version 2

**DOI:** 10.5256/f1000research.12397.r22240

**Published:** 2017-04-26

**Authors:** Jean-Paul J. Gonzalez, Juergen Richt

**Affiliations:** 1 Center of Excellence for Emerging and Zoonotic Animal Diseases (CEEZAD), College of Veterinary Medicine, Kansas State University, Manhattan, KS, USA; 2 Center of Excellence for Emerging and Zoonotic Animal Diseases (CEEZAD), College of Veterinary Medicine, Kansas State University, Manhattan, KS, USA

I agree with the version 2 of the manuscript and I approved it for publication.

We have read this submission. We believe that we have an appropriate level of expertise to confirm that it is of an acceptable scientific standard.

# Peer review: Referee response for version 1

**DOI:** 10.5256/f1000research.12169.r21389

**Published:** 2017-04-18

**Authors:** Paola Barato

**Affiliations:** 1 Veterinary Pathobiology Group, Laboratory of Veterinary Pathology, Institute of Biotechnology, National University of Colombia, Bogotá, Colombia; 2 CEO and Scientific Director, CORPAVET, Bogotá, Colombia

In general, it is an interesting and well written opinion article. Some comments:

 TITLE: Okay.

 ABSTRACT: Okay.

 INTRODUCTION:

 1. I consider it to be desirable to include in the introduction the name of the virus (species) which causes yellow fever.

 2. Please review the punctuation of this sentence: .."In Asia, but also Europe and North America, Nevertheless, potential spreads beyond the borders of the endemic countries is a matter of global concern."

 RECENT OUTBREAKS

 3. Because this section is referring only to information outside of Americas, I respectfully suggest modified the subtitle as: RECENT OUTBREAKS OUTSIDE OF AMERICAS

 THE CONCERN RAISED FROM BRAZIL

 In the introduction was stated.."the lack of efficient health policies", however in the development of this idea in this section there is very little (a sentence) about what are the Brazilian health policies for yellow fever or for vector-borne disease.

 This information could be very useful to go in deep to discussion about the "lack of efficient health policies"

 CONCLUSIONS

 Again, the statement "the lack of efficient health policies" needs a deeper discussion in the previous section to be included in the conclusions.

 I have read this submission. I believe that I have an appropriate level of expertise to confirm that it is of an acceptable scientific standard, however I strongly recommend to include the comments outlined above.

I have read this submission. I believe that I have an appropriate level of expertise to confirm that it is of an acceptable scientific standard.

# Response

**Published:** 2017-04-19

**Authors:** Alfonso Rodriguez-Morales

**Affiliations:** Universidad Tecnológica de Pereira, Colombia

Dear Dr. Barato

Thank you very much for your valuable assessment and comments. Regard the specific comments:

**INTRODUCTION:**

**1. I consider it to be desirable to include in the introduction the name of the virus (species) which causes yellow fever.**

Agree, we will include it.

**2. Please review the punctuation of this sentence: .."In Asia, but also Europe and North America, Nevertheless, potential spreads beyond the borders of the endemic countries is a matter of global concern."**

Agree, we will review it.

**RECENT OUTBREAKS**

**3. Because this section is referring only to information outside of Americas, I respectfully suggest modified the subtitle as: RECENT OUTBREAKS OUTSIDE OF AMERICAS**

Thanks for your comment, we will modify it.

**THE CONCERN RAISED FROM BRAZIL**

**In the introduction was stated.."the lack of efficient health policies", however in the development of this idea in this section there is very little (a sentence) about what are the Brazilian health policies for yellow fever or for vector-borne disease.**

**This information could be very useful to go in deep to discussion about the "lack of efficient health policies"**

We will include information about the Brazilian health policies for yellow fever and vector-borne diseases.

**CONCLUSIONS**

**Again, the statement "the lack of efficient health policies" needs a deeper discussion in the previous section to be included in the conclusions.**

Ok, we will go deeper in the discussion regarding that point.

# Peer review: Referee response for version 1

**DOI:** 10.5256/f1000research.12169.r21529

**Published:** 2017-04-04

**Authors:** Jean-Paul J. Gonzalez, Juergen Richt

**Affiliations:** 1 Center of Excellence for Emerging and Zoonotic Animal Diseases (CEEZAD), College of Veterinary Medicine, Kansas State University, Manhattan, KS, USA

**Title:**  Although the title is appropriate and clearly leads to the risk of YF re-emergence in the Americas, the manuscript is more centred to South and eventually Central Americas. Also, for this matter (i.e. maintain the title), the authors could emphasize strongly on the risk of imported cases in temperate zone (i.e. Central and North Americas) during the boreal summer and *Aedes* spp. activity in Central-North America (e.g. as it is documented elsewhere for Dengue virus - New Mexico or Texas – and, Airport malaria in the US).

**Abstract:** “Angola” appears at first, also if the authors want to focus on the Americas, it will be better, in my opinion, to have a short sentence at the end of the abstract that focus on imported risk from endemic area outside of Americas (i.e. Africa). 

 The authors wrote “despite high YF vaccination coverage”, this is not accurate: Indeed, in many areas and populations worldwide, YF vaccination coverage is discouraging low for years (e.g. Nigeria). This needs to be clear: YF vaccine is certainly the best live attenuated vaccine among all, the less expensive and the first of its kind, consequently there is no reasons today – except politics and funding allocation - to have the people of endemic areas not yet entirely immunized with a real 100% vaccine coverage.

**Introduction:** For the reader, CFR needs to be expressed as a number of a general historical consensus.

 Needs also to document the historical dimension of multiple consistent re-emergence of Yellow fever since it discovery beside the excellence of the vaccine(s). While frequency and size of outbreaks are recently (a decade ago) increasing.

**Recent outbreaks**: China emergence needs to be more specific (i.e. risk) from where (climatic zone) these imported cases were observed.

**“Concern raised from Brazil”**:

- From the general title or this of such chapter section, one is misleading “Americas (title) or Brazil (this section)”? I suggest something like: “From Brazilian experience, a concern of YF risk for the Americas”
- Line 3: “epicentre:” this needs to be more precise geographically or the sentence clearly linked to the following one, starting by “Indeed, …“
- Second section, line 4: “a long history of high YF vaccination coverage”, I am not sure this is applicable to Minas Gerais’s remote areas, at least for “long history”. The lack of YF vaccination coverage was raised several times by the Brazilian provincial health authorities back in the early 2000s, unable to reach the remote western zones of the province.
- Section 3, line 3, top of the page:  to be politically correct we do not use anymore “Latin America” but “South America”.

 

***Conclusions***: Main concerns, regarding the YF risk of emergence/re-emergence, seems to be missing:

- Vaccine: 1/ The recent outbreaks and the lack of Yellow fever vaccine stock piling (WHO). This needs to be strategized (YF vaccine availability) by the country health authorities and international community. 2/ Also the lifelong protection of the vaccine, its inocuity, and the reduction by 1/10 of the immunity dose are new and of extremely high importance (i.e. for the public & public health).
- Biosurveillance needs to be stressed: Mosquito biosurveillance is an important issue to control the epidemic risk, also *Haemagogus *and *Sabethes are* specific for South America and have well studied, the risk and ability of *Aedes albopictus *(expansion) to transmit the virus in the Americas needs to be assessed and an entomological priority set up when needed (i.e. Public health priority in at risk areas).
- Trans-border risk. Ultimately traveler’s from/to endemic areas need to be covered by a mandatory international certificate of vaccination to protect the borders (trans-border risk).
- The long time mystery of the absence of YFV in South East Asia can be also stressed in term of global risk.

We have read this submission. We believe that we have an appropriate level of expertise to confirm that it is of an acceptable scientific standard, however we have significant reservations, as outlined above.

# Response

**Published:** 2017-04-04

**Authors:** Alfonso Rodriguez-Morales

**Affiliations:** Universidad Tecnológica de Pereira, Colombia

Dear Drs. Gonzalez and Richt

 Thanks for you valuable comments. The first thing that should be pointed out is that this is not a Review Article, is a short Opinion Article. Nevertheless, we fully agree to carefully revise our manuscript for a new version (Version 2), considering all the comments you made and based on that correct it accordingly base on each of them.

 Regard your comments:

**Title: Although the title is appropriate and clearly leads to the risk of YF re-emergence in the Americas, the manuscript is more centred to South and eventually Central Americas.**

 Yes, certainly this was focused on the concern of expansion in Latin America beyond Brazil where currently is an epidemic situation, where, since the beginning of the outbreak in December 2016 up to 29 March 2017, there were 1,987 cases of yellow fever reported (574 confirmed, 926 discarded, and 487 suspected under investigation), including 282 deaths (187 confirmed, 24 discarded, and 71 under investigation). The case fatality rate (CFR) is 33% among confirmed cases.

**Also, for this matter (i.e. maintain the title), the authors could emphasize strongly on the risk of imported cases in temperate zone (i.e. Central and North Americas) during the boreal summer and Aedes spp. activity in Central-North America (e.g. as it is documented elsewhere for Dengue virus - New Mexico or Texas – and, Airport malaria in the US).**

 We agree with this comment. This will be definitively included in our new version.

**Abstract: “Angola” appears at first, also if the authors want to focus on the Americas, it will be better, in my opinion, to have a short sentence at the end of the abstract that focus on imported risk from endemic area outside of Americas (i.e. Africa).**

 Fully agree, we will change the abstract according those considerations.

**The authors wrote “despite high YF vaccination coverage”, this is not accurate: Indeed, in many areas and populations worldwide, YF vaccination coverage is discouraging low for years (e.g. Nigeria). This needs to be clear: YF vaccine is certainly the best live attenuated vaccine among all, the less expensive and the first of its kind, consequently there is no reasons today – except politics and funding allocation - to have the people of endemic areas not yet entirely immunized with a real 100% vaccine coverage.**

 We would rephrase that, in order to make clear that although in some areas of some countries at risk, there is a high YF vaccination coverage, there are many areas and populations worldwide, where that is low for years (e.g. Nigeria).

**Introduction: For the reader, CFR needs to be expressed as a number of a general historical consensus.**

 We will explain more about the CFR historical reports.

**Needs also to document the historical dimension of multiple consistent re-emergence of Yellow fever since it discovery beside the excellence of the vaccine(s). While frequency and size of outbreaks are recently (a decade ago) increasing.**

 Ok. We will also comment on this, according to your recommendation.

**Recent outbreaks: China emergence needs to be more specific (i.e. risk) from where (climatic zone) these imported cases were observed.**

 Ok. Now is more detailed available information about it, then we will address this in the revised version.

**“Concern raised from Brazil”:**

**From the general title or this of such chapter section, one is misleading “Americas (title) or Brazil (this section)”? I suggest something like: “From Brazilian experience, a concern of YF risk for the Americas”**

 Well, the concern is for Americas, Brazil is already with epidemics. Then, given that, we will change the title of section to “From Brazilian experience, a concern of YF risk for the Americas”.

**Line 3: “epicentre:” this needs to be more precise geographically or the sentence clearly linked to the following one, starting by “Indeed, …“**

 Ok, we will correct it.

      

**Second section, line 4: “a long history of high YF vaccination coverage”, I am not sure this is applicable to Minas Gerais’s remote areas, at least for “long history”. The lack of YF vaccination coverage was raised several times by the Brazilian provincial health authorities back in the early 2000s, unable to reach the remote western zones of the province.**

 Agree, we will make such clarification.

      

**Section 3, line 3, top of the page:  to be politically correct we do not use anymore “Latin America” but “South America”.**

 Well, that is not really accurate, both terms are correct. But Latin America includes both Central and South America. You can consult any reference and you will realize this. South America is not exchangeable to Latin America, with this you will be excluding Central America and Mexico.

**Conclusions: Main concerns, regarding the YF risk of emergence/re-emergence, seems to be missing:**

**Vaccine:**

**1/ The recent outbreaks and the lack of Yellow fever vaccine stock piling (WHO). This needs to be strategized (YF vaccine availability) by the country health authorities and international community.**

 This will be included in our new revised version.

 2/ Also the lifelong protection of the vaccine, its inocuity, and the reduction by 1/10 of the immunity dose are new and of extremely high importance (i.e. for the public & public health).

 This too.

    

**Biosurveillance needs to be stressed: Mosquito biosurveillance is an important issue to control the epidemic risk, also Haemagogus and Sabethes are specific for South America and have well studied, the risk and ability of Aedes albopictus (expansion) to transmit the virus in the Americas needs to be assessed and an entomological priority set up when needed (i.e. Public health priority in at risk areas).**

 We will add comments about this.

**Trans-border risk. Ultimately traveler’s from/to endemic areas need to be covered by a mandatory international certificate of vaccination to protect the borders (trans-border risk).**

 Ok, agree. We will include comments about this.

      

**The long time mystery of the absence of YFV in South East Asia can be also stressed in term of global risk.**

 Ok, we will make also comments on this.
